# Cake, Checkups, and Captain Starlight: Evaluating the Cherbourg Third Birthday Party Health Initiative for Children in Rural Australia

**DOI:** 10.1111/ajr.70082

**Published:** 2025-08-07

**Authors:** Claire Treadgold, Erika Fortunati, Rob Doyle, Jo Dann, Aunty Kerrie Doyle

**Affiliations:** ^1^ Starlight Children's Foundation Australia Sydney New South Wales Australia; ^2^ Faculty of Medicine University of New South Wales Sydney New South Wales Australia; ^3^ Indigenous Health, School of Medicine Western Sydney University Sydney New South Wales Australia

## Abstract

**Objective:**

Aboriginal and Torres Strait Islander children, particularly those in remote Australia, face disproportionately higher rates of preventable health conditions and disability. Early intervention is considered particularly important for this demographic, but previous attempts have had limited success.

**Design:**

In response to a need identified by the Cherbourg Health Service, Starlight Children's Foundation Australia (Starlight) partnered with them to host a unique “third birthday party” event in Cherbourg, Queensland.

**Setting and Participants:**

The event aimed to provide health checks and a culturally sensitive, positive healthcare experience for three‐year‐old children and the Cherbourg community, incorporating key health service providers and Starlight “Captains” to facilitate the health checks and activities/games. Main outcome measure(s): To evaluate the third birthday party health initiative, the main outcome measures were the strengths and future considerations and improvements of the event.

**Result:**

The quantitative and qualitative data highlighted the event's success in promoting an effective and positive community‐led healthcare experience by employing a unique, prevention‐focused methodology, with benefits extending from the community to health staff and students.

**Conclusion:**

Overall, the Cherbourg third birthday party serves as a model for culturally appropriate early health interventions in Australia, offering valuable insights to enhance healthcare promotion, access, and engagement for Indigenous children and communities.

**Summary**

- What this paper adds and what is already known on this subject:
  ○ Aboriginal and Torres Strait Islander children in remote Australia face high rates of preventable health conditions, yet early‐intervention efforts often struggle with engagement and effectiveness. While culturally responsive, community‐led healthcare initiatives are essential, participation in health checks for 0–4‐year‐old First Nations children remains low. This study introduces a novel community‐driven “third birthday party” model that successfully integrated health checks into a celebratory, play‐based event, fostering a positive healthcare experience and addressing generational concerns. The approach increased participation, strengthened community health service relationships, and enhanced cultural competency among healthcare staff and students. Findings highlight the value of community involvement and provide insights for scaling similar culturally appropriate interventions in other Indigenous communities.

## 1 Introduction

Aboriginal and Torres Strait Islander children experience significant health disparities compared to non‐Indigenous Australian children [1, 2]. This health gap is characterised by an increased prevalence of preventable disease, lower life expectancy, and higher rates of mortality [3, 4, 5]. Notably, the overall burden of disease for Indigenous Australians is 2.3 times the rate of non‐Indigenous Australians [6]. Moreover, Indigenous children living in remote Australia experience higher rates of disease burden and lower life expectancy compared to those in non‐remote areas [2, 6]. Common health concerns among Indigenous children in Australia include oral, dental, and hearing health; immunisation; diet and nutrition; chronic health conditions; speech pathologies; and disability [5, 6, 7].

These disparities, stemming from historical and systematic inequalities and limited access to quality healthcare [8, 9, 10], call for comprehensive, culturally responsive, early‐intervention public health initiatives for Indigenous children living in remote Australia. The early intervention and detection of health issues are critical factors in reducing the burden of disease in Indigenous children, which are set to have cascading beneficial effects on future generations, endeavouring to “close the gap” between Indigenous and non‐Indigenous children's health outcomes in Australia [2, 11].

Cherbourg is a small, rural town in Queensland, Australia, with a primary health service known as the Cherbourg Health Service. The Cherbourg Community Health Service has identified that several families engage in their service to have their children immunised at 18 months, but tend to delay further medical visits until the children reach school age. However, it is well established that health and developmental checks between the formative years of two and four are crucial to identify and address preventable diseases and developmental disorders early to avoid unnecessary health consequences for the child [11, 12].

Starlight Children's Foundation (Starlight) is a not‐for‐profit organisation that works with health professionals to positively reframe the healthcare experience of children and young people. Many of the programs involve “Captain Starlight”, professional, costumed performers specifically trained to engage with children. Starlight has a strong history of partnering with remote Aboriginal and Torres Strait Islander communities through the “Healthier Futures Initiative”, which is a partnership with local communities and health teams that focuses on the identification and treatment of preventable health conditions. With invitations from the communities, Starlight Captains visit them, aiming to help mitigate health‐related anxiety and increase participation in healthcare by providing positive distraction through song, dance, games, and storytelling for children.

This paper will describe the third birthday party event, including its methodology, benefits, considerations for future iterations, and satisfaction as described by children, their families, healthcare professionals, and Starlight Captains and team members.

## 2 Methods

Following the successful involvement of Starlight Captains in clinical visits to Cherbourg and other communities with the Queensland Health programme “Deadly Ears” (a statewide initiative aimed at improving ear health for Aboriginal and Torres Strait Islander children), a desire by the clinical team to create earlier referral pathways resulted in the birthday party concept. The concept, which leaned into Starlight's expertise in engaging children, was expanded and aimed to address broader concerns around the Cherbourg community's younger children's health and developmental outcomes. Representatives from Starlight met with Cherbourg's already well‐established Health Advisory Group (HAG), which included local Elders as well as health professionals and local community representatives. An ongoing meeting was set up with the HAG to organise the event, and it expanded to include representatives from Queensland Health and Southern Queensland Rural Health. This group oversaw the planning of the birthday party event, consulting closely with other Elders and the community about the proposed idea and seeking input about participants, content, timing, risk management and promotion.

All local children in Cherbourg aged three or turning three in 2021 were invited to attend a joint third birthday party event hosted near the local medical service in Cherbourg. The Community Health Nurse was able to use the local area health data to create a list of those in the relevant age group and handed out invitations to every child and their family. Several health service providers participated in the event, including representatives from hearing, physiotherapy, occupational therapy, dietetics, speech, optometry, child health, and dental health services. Starlight Captains were also invited to facilitate the event, greeting children as they arrived, providing entertainment, helping the children feel at ease, and assisting the health teams in delivering health checks.

Posters and signs were developed and distributed to promote the event around Cherbourg, and radio announcements were coordinated a month before the event, with local radio being a key communication asset in the community. Other local organisations and community groups (such as the local childcare facility) were advised and invited to participate. It was made clear that all siblings were also welcome to be involved on the day.

**FIGURE 1:**
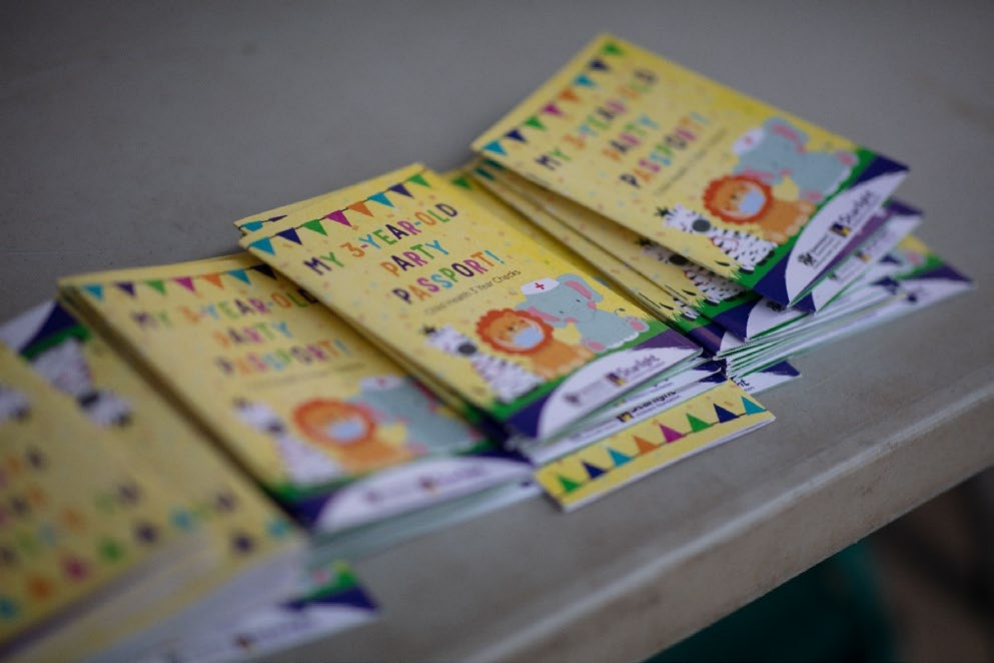
The Cherbourg third birthday passport.

**FIGURE 2:**
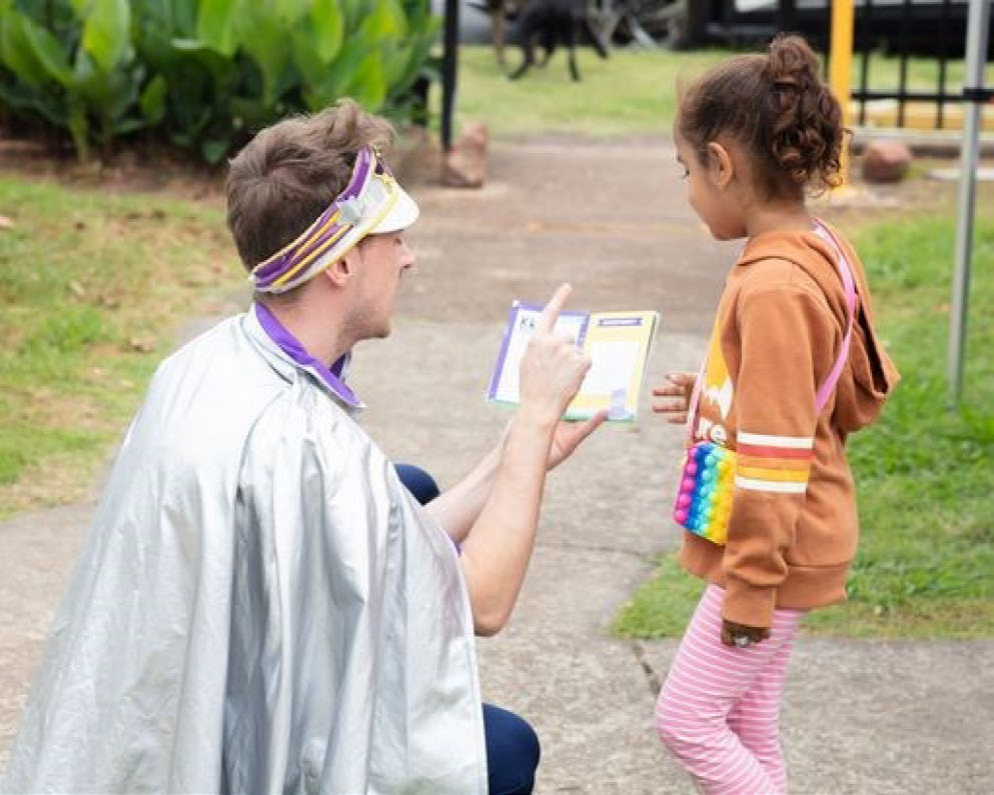
Starlight Captain engaging a child using the health passport.

**FIGURE 3:**
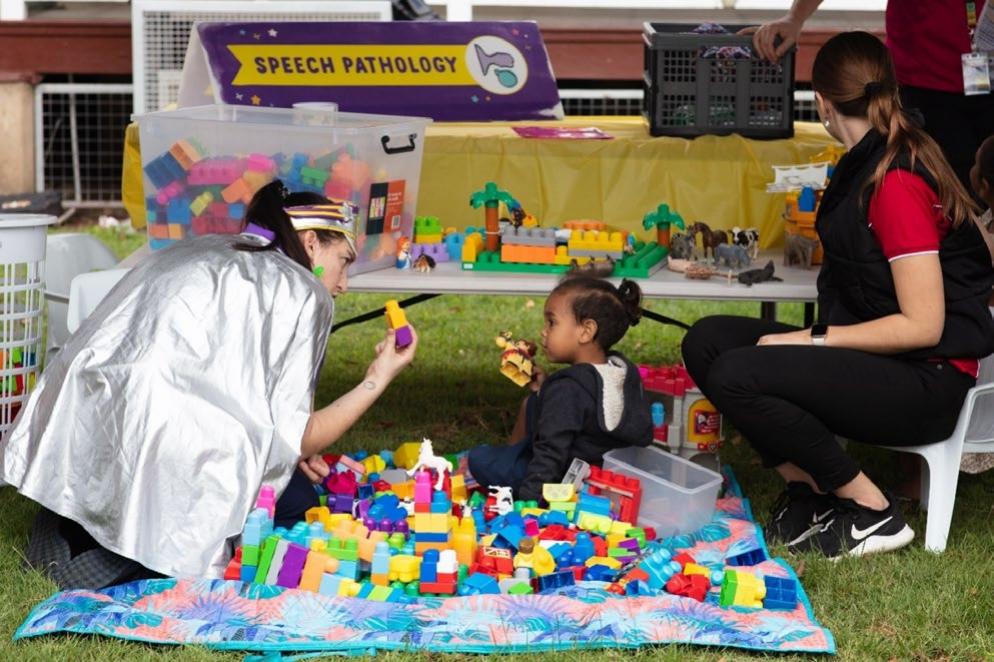
Starlight Captain (left) helping facilitate a health check, engaging the child through play.

A passport‐sized booklet was developed by Starlight (herein referred to as the “health passport”), with the first page including the child's name and a Polaroid photo of themselves taken on the day of the event, followed by a page dedicated to each of the health stations (Figure 1). Once a child attended a health station, they would be assessed by the health team and receive a stamp on their health passport to indicate their attendance. During the assessment, health professionals would note anything of concern and coordinate the necessary follow‐up checks (Figures 2 and 3). “Goodie bags” with age‐appropriate toys and games were also organised for the children and their siblings after they had attended all health stations.

A Welcome to and Acknowledgement of Country occurred at the beginning of the party, with culturally relevant music playing during the event. Other event management items, e.g., tables, party decorations, and catering with locally available food and celebratory birthday cakes, were also arranged.

The evaluation design was a collaboration between Starlight and the Aboriginal Health and Wellbeing Clinical Advisory Group from Western Sydney University to develop a culturally appropriate and low‐burden approach to the evaluation. Based on extensive experience in health and Aboriginal communities, the team led by Professor Aunty Kerrie Doyle provided recommendations to ensure the input of children and community members was appropriately included.

**FIGURE 4:**
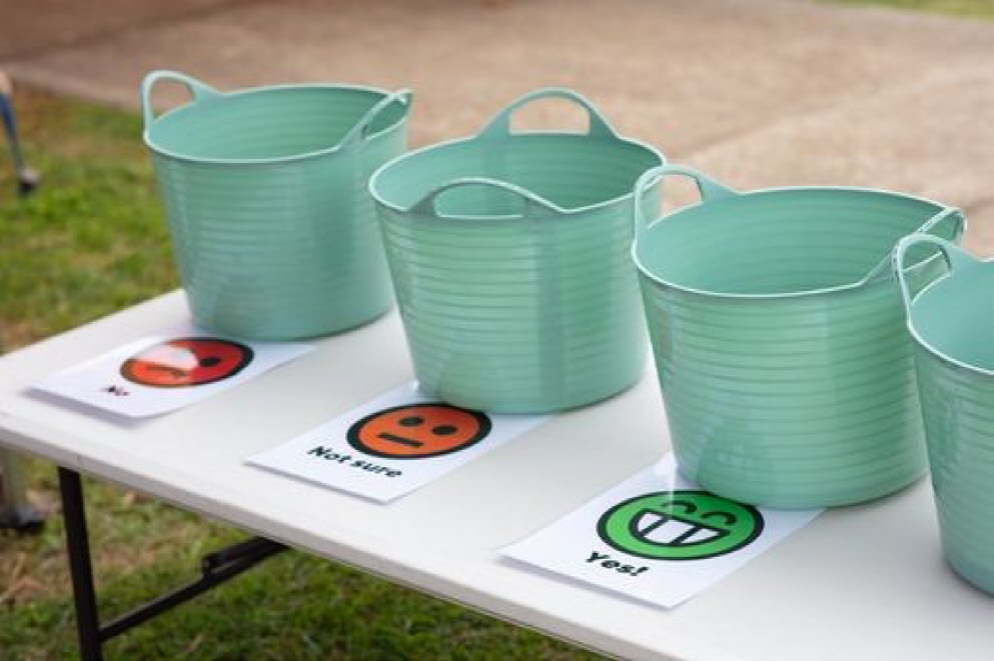
Evaluation method for collecting child satisfaction.

**FIGURE 5:**
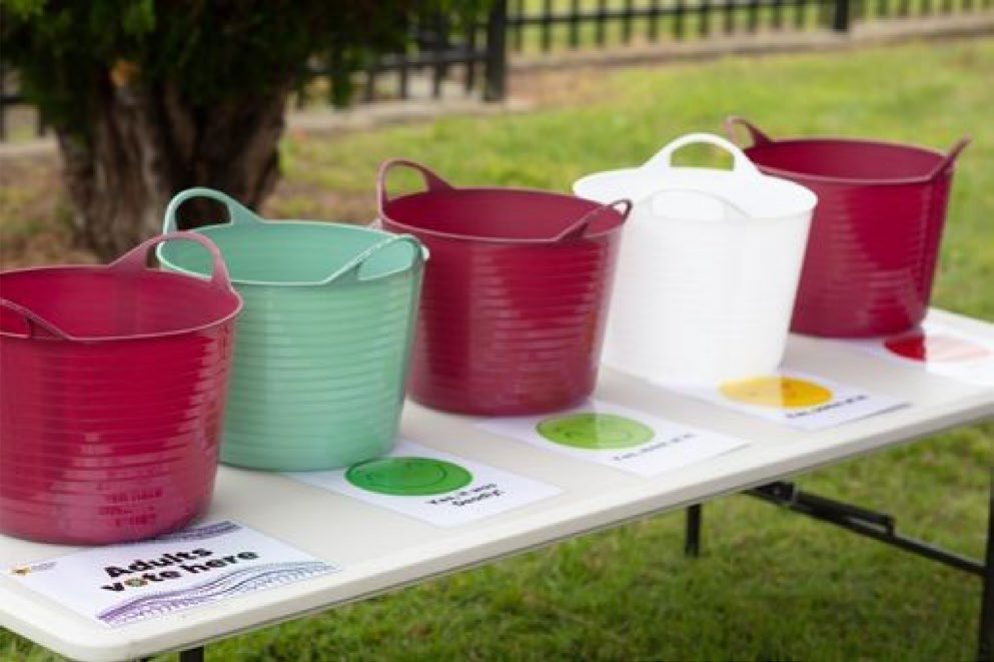
Evaluation method for collecting adult satisfaction.

Quantitative data were collected by gathering satisfaction ratings from children and caregivers using two sets of feedback buckets: three buckets with yes/no/unsure options for children (Figure 4), and five buckets for adults with five emojis to indicate satisfaction levels (Figure 5). Upon leaving the party, children and caregivers received tokens and were encouraged to place them in the bucket that best represented their satisfaction. After the event had ended, the tokens in each bucket were counted and tallied for analysis (Tables 1 and 2).

**TABLE 1:** Number of children's votes in each survey bucket.

“Yes”	“Not sure”	“No”
31	2	2

**TABLE 2:** Number of caregiver votes in each survey bucket.

“Yes, it was deadly!”^a^	“Yes, most of it”	“Some of it”	“A little of it”	“No, none of it”
25	0	0	0	0

^a^ “Deadly” has positive connotations in Aboriginal Australian culture.

Qualitative data in the form of semi‐structured interviews with the health professionals, Starlight team members, and Starlight Captains were collected via phone or video meeting by R.D. The questions were based on their experience with the third birthday party, suggestions for improvement, and the perceived impact of the event on the community (Supporting Information). Interviews were audio‐recorded, de‐identified, and transcribed verbatim. Thematic analysis was conducted using NVivo Version 12.0 by E.F. and R.D. following the well‐known Braun and Clarke's six‐step method to thematic analysis [13]. E.F. and R.D. both coded the verbatim transcripts independently to reduce researcher bias. E.F. then developed the thematic framework by iteratively grouping codes into potential themes, which were refined and finalised into the individual themes presented in this paper. The final coding framework was determined by E.F. based on the independent coding analysis conducted by both researchers. We received ethics approval from Western Sydney University (HREC/2020/WSU/H11372).

## 3 Results

Approximately 80 children, accompanied by caregivers, attended the event, including individual groups of children and caregivers and local kindergarten attendees. In total, 35 children and 25 adults participated in the survey, answering “Did you enjoy the birthday party today?” (Tables 1 and 2).

**FIGURE 6:**
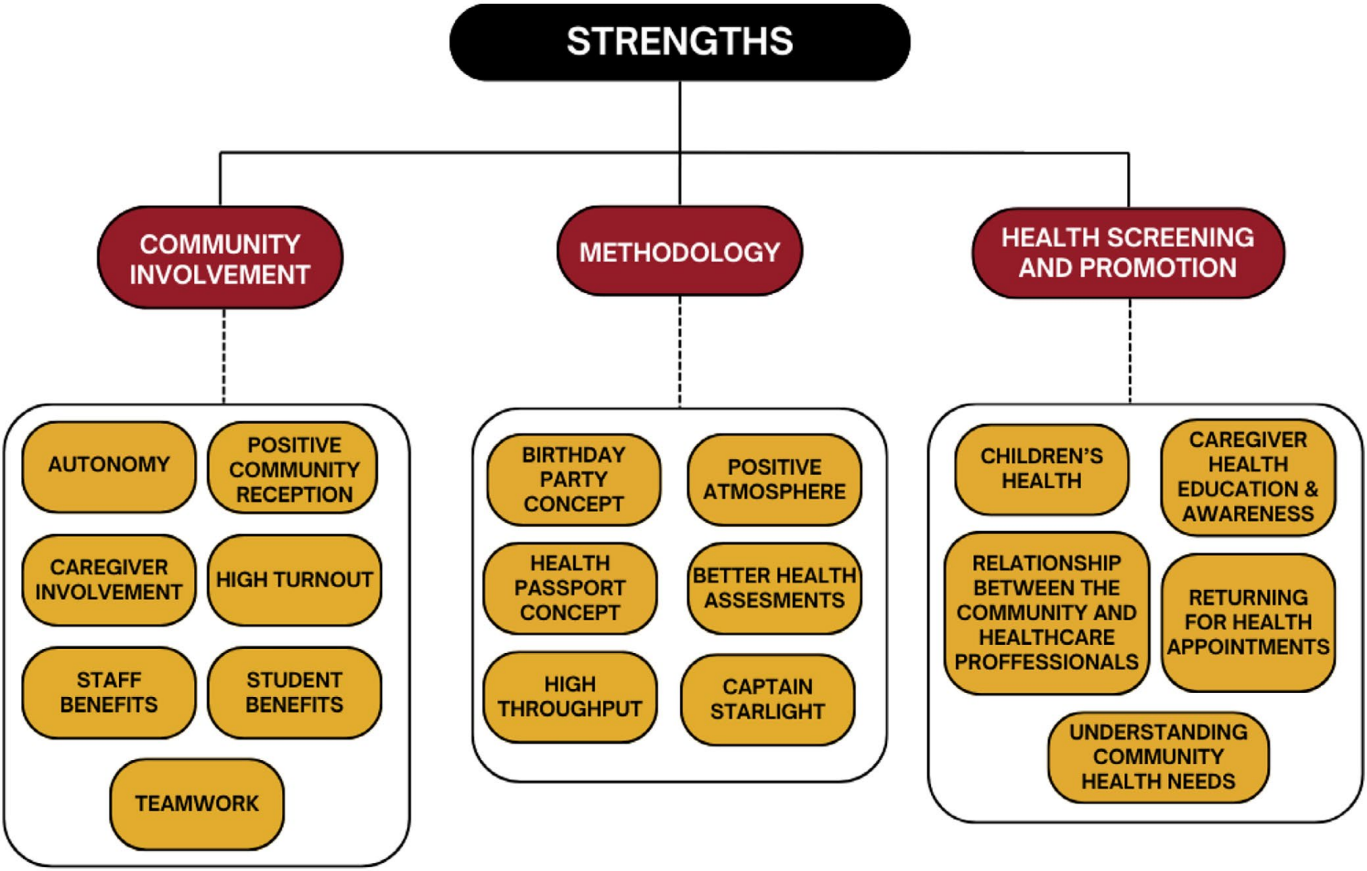
Strengths of the Cherbourg third birthday party.

**FIGURE 7:**
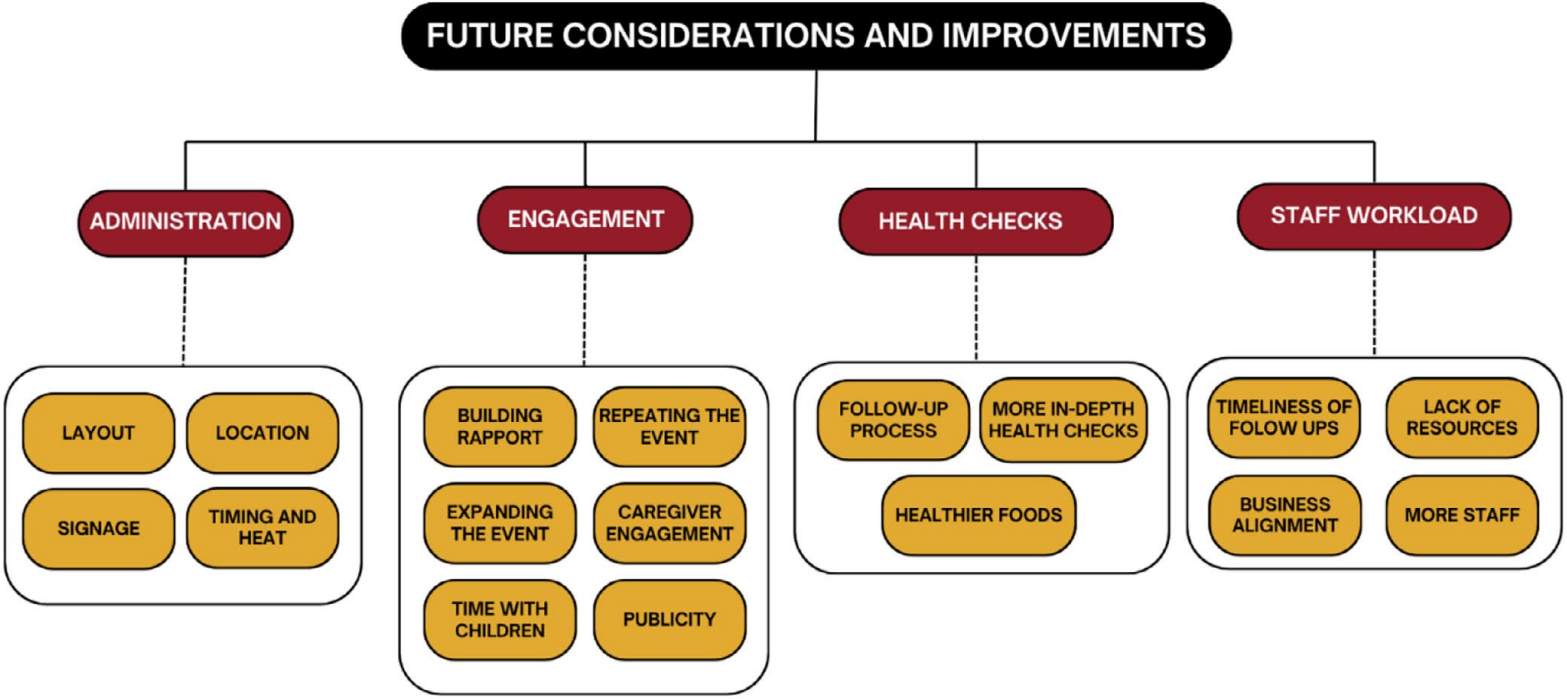
Future considerations and improvements for the Cherbourg third birthday party.

Specialists in hearing, optometry, physiotherapy, occupational therapy, dietetics, speech, child health, and dental health attended the event. Of these, 15 were interviewed by R.D. Starlight team members and Captains who were involved in the planning and coordination and/or attended the event, were also interviewed. In total, 10 interviews were conducted with health professionals, and five were conducted with Starlight team members and Captains. From these interviews, the need and cultural considerations for the event, strengths of the event, and future considerations and improvements for future versions of the event were discussed. There were three emergent themes under the strengths of the event, and four emergent themes under future considerations, which were subdivided into 18 and 17 subthemes for the strengths and future considerations/improvements, respectively (Figures 6 and 7).

### 3.1 Need and Cultural Considerations for the Event

Interviewed participants agreed on the event's necessity, underscoring the concern that children in Cherbourg and rural Australia are “falling through the cracks”, missing crucial health checks before kindergarten, leading to undiagnosed and worsening health conditions. They identified a lack of health education, placing a significant burden on parents, especially considering Cherbourg's young population. It was hoped that an event like the third birthday party could help identify and treat at‐risk children, improving their health outcomes. Participants also highlighted the need for more community celebrations in Cherbourg, especially considering the community's history and the imperativeness of strong community connections and cultural sensitivity. Illustrative quotes can be found in Table S1.

### 3.2 Strengths of the Event

Using a thematic analysis approach, the event's strengths can be broadly divided into three themes: community involvement, methodology, and health screening and promotion, which are further divided into 18 subthemes (Figure 6). Illustrative quotes can be found in Table S2.

#### 3.2.1 Community Involvement

The opt‐in approach gave the community autonomy to choose their participation, creating a positive reception from the community. The community's engagement, including the Elders' and community representatives' involvement, supported a sense of unity towards the common goal of supporting children's health in Cherbourg, which was perceived as contributing to the day's success, marked by a high turnout and caregiver involvement. Participants found the event personally rewarding as it provided an opportunity to connect and network with the community and other local health professionals, increasing awareness of other health service offerings. Some health professionals brought students to help with the event, who also benefited from interacting with Indigenous and remote communities and applying their skills outside of a traditional learning environment. Many attributed the day's success to the teamwork between the community, facilitators, and health professionals.

#### 3.2.2 Methodology

Participants conceptualised the third birthday party as a holistic, welcoming, and fun way to conduct community health checks. Moving health checks outside the clinic and into a more familiar environment created a relaxed atmosphere. The mix of fun activities to health checks was seen as balanced, providing fun while offering valuable health assessments and fostering a positive atmosphere. Interviewees thought that the “health passport” concept, with children receiving stamps at every station, topped off with a goodie bag, was a good incentive for children and families to engage with multiple health stations. The Starlight Captains were seen as a significant positive influence on the event's success, effectively easing the children's nerves and supporting health professionals in conducting their assessments. The physiotherapy and speech‐language therapy professionals noted that the event's informal setting was conducive to better health assessments than in a clinic environment; in this relaxed outdoor context, children might have displayed more “natural” behaviours e.g., running around outside and playing games, allowing healthcare professionals to observe abilities that might not be evident during formal clinical appointments where children may be more nervous or guarded. Some participants noted that the methodology facilitated a high throughput of health screenings, overcoming the typically long in‐clinic wait times.

#### 3.2.3 Health Screening and Promotion

Participants perceived the event as providing health benefits for children, as it allowed them to screen many children across key health disciplines, identifying those needing follow‐up earlier than they would without the event. The event was also perceived as benefiting caregivers in terms of increasing their awareness of health service offerings for children and understanding the expectations of children's health and development according to their age.

Some interviewees acknowledged that while they did not identify any children needing follow‐up health support, they still saw value in having face‐to‐face contact with the community and promoting positive health behaviours and their services. Importantly, the event was viewed as providing a corrective experience to improve the relationship between the Cherbourg community and health professionals by providing a safe space that transforms medical visits from intimidating and scary experiences to more fun and comfortable interactions. As a result of this event, participants received follow‐up requests and reported increased willingness from families for future event iterations and other health appointments. While not all health stations received formal referrals from the event, some participants said the event helped them better understand the community's health needs, supporting their ability to provide future care.

### 3.3 Future Considerations and Improvements

The event's future considerations and improvements are divided into four themes: administrative considerations, engagement, health checks, and staff workload, further divided into 17 subthemes (Figure 7). Illustrative quotes can be found in Table S3.

#### 3.3.1 Administrative Considerations

Participants generally found the event well‐organised but suggested improvements in layout, including better spacing between stations and clearer signage to reduce crowding and confusion. Some recommended hosting it in a more central location to attract more foot traffic. Timing was a concern, with midday heat potentially reducing attendance. While water bottles, a mobile cold room, and marquees were appreciated, participants recommended an earlier start time and scheduling the event during the year's cooler months.

#### 3.3.2 Engagement

Participants acknowledged that while the event facilitated positive community connections, developing rapport will require more positive experiences over time. In fact, several participants recommended that the event become an annual occurrence to increase awareness and attract more attendees. Many recommended expanding the event to other rural areas, given the similar health concerns.

To improve the event's long‐term impact, participants suggested focusing more on supporting and engaging caregivers, e.g., providing take‐away booklets and health strategies, and encouraging health professionals to ask caregivers more in‐depth questions about children's health and development, especially as they believed caregivers significantly influence children's health. Some proposed offering caregivers health checks and tokens of appreciation to increase their engagement. Participants also suggested that a more consistent flow of children to each station would have reduced overcrowding and allowed health professionals more time for quality assessments. Lastly, participants frequently recommended increased publicity and a broader multimedia strategy to boost attendee numbers, with a more targeted approach for “hard‐to‐reach” community members.

#### 3.3.3 Health Checks

Participants emphasised the need for a more streamlined follow‐up referral process, citing challenges in tracking and responding to referrals. Some also recommended conducting more in‐depth assessments at their stations, while others noted the limited time spent with children hindered their ability to conduct a thorough assessment. However, one station preferred offering general health support due to administrative constraints. The dietitian station suggested healthier food options at the event, e.g., carrot cake rather than traditional birthday cake, to align with their station's health messages.

#### 3.3.4 Staff Workload

Many participants noted that the event negatively impacted their workload, with concerns about timely referral follow‐up due to the lack of staff capacity in the region. Additionally, limited resources and competing clinical priorities in rural Australia also meant some staff left early or were not able to attend at all. Many participants recommended involving more students and non‐clinical staff, especially in the project planning and set‐up process, to help mitigate the effect of understaffing while supporting students' career development.

## 4 Discussion

### 4.1 Quantitative Data

Most children (31/35) enjoyed the birthday party, as indicated by their “yes” votes. Caregivers unanimously enjoyed the event, with 100% voting “Yes, it was deadly!” (note that “deadly” has positive connotations in Aboriginal Australian culture). Overall, feedback from both children and caregivers was highly positive, indicating strong satisfaction.

### 4.2 Need and Cultural Considerations

The data underscore the real concern of children missing critical health checks before kindergarten, leading to missed diagnoses and the incidence and worsening of preventable health conditions. Indigenous children in remote Australia face some of the country's worst health, experiencing exceptionally high rates of hospitalisation, emergency department visits, and disabilities [2, 5, 6]; one in five Aboriginal and Torres Strait Islander children live with a disability, including sensory (12%), cognitive (9%), physical (5%), and psychosocial disability (4%) [2].

The New South Wales first 2000 days framework reports that 42% of Aboriginal and Torres Strait Islander children are at risk of developmental delay at school entry, indicating that these children could have missed the benefits of early intervention [14]. Furthermore, limited health education for Indigenous Australians, particularly in remote areas, further contributes to poor health outcomes [15, 16, 17, 18]. Health promotion initiatives to address the health and health education of Indigenous children have been somewhat unsuccessful, with only 26% of Indigenous children aged 0–4 receiving government‐subsidised health checks in 2020–2021 [15, 19]. Best practice for engaging this population encourages adapting care models for rural areas [20]. This calls for novel and culturally sensitive approaches to meet the specific health needs of Indigenous Australian children.

Cherbourg, originally Baramah, rests on the ancestral lands of the Wakka Wakka Aboriginal people of Australia [21]. Cherbourg's history reflects systematic injustices dating back to European settlement in the mid‐19th century, causing forced family separations, cultural assimilation, and land loss for the Indigenous population [21, 22]. This led to profound health consequences for Indigenous people, including inadequate living conditions, prevalent disease, limited access to healthcare, and high mortality rates [21, 22]. Consequently, distrust in institutionalised health systems has followed [9, 22]. Therefore, the importance of trust and respect between community members and the healthcare system cannot be understated for culturally safe and effective healthcare delivery [23, 24].

### 4.3 Community Involvement

An opt‐in, community‐led approach proved successful in fostering strong community involvement, evident in the high turnout, positive reception, children's satisfaction, and caregiver engagement. This approach challenges conventional top‐down health strategies by empowering community members to make their own decisions regarding their health, fostering autonomy [25]. Incorporating cultural practices and traditions, e.g., traditional song and dance, local food, speeches from Elders, etc., not only elevates community representative voices and representation but also recognises the cultural nuances of individual communities and the importance of building meaningful and trusting relationships with community members [23].

The data suggest that the event also benefited clinical team members and students. The event allowed health professionals and students to forge meaningful connections with the community and gain valuable hands‐on experience in an Indigenous, rural Australian setting. This supports clinicians' and students' career development as culturally sensitive practitioners, with the hope of breaking the systemic lack of Indigenous cultural competency in the Australian healthcare system [2, 24]. Strengthening rapport and trust between clinicians and patients is also known to support quality healthcare for Indigenous Australians [24]. Moreover, the event provided an opportunity for team members to network with other health services, thereby broadening their understanding of available health services and their scope of potential referrals, which is important due to the professional isolation and increased scope of practice experienced by health professionals working in rural Australia [26, 27]. Thus, this event may improve the interconnectedness of the health system in and around Cherbourg, ultimately benefiting the community's health.

### 4.4 Methodology

While this is not the first “health passport” initiative used for a health promotion programme, to our knowledge, this is the first of its kind in the context of facilitating health screening and promotion for Indigenous children's health in rural Australia.

Incorporating gamification into health programmes targeted at children—albeit a relatively new phenomenon—has proven successful in emerging studies [28, 29]. By illustrating the event's goals and their relevance, nudging users through guided paths (in this context, incentivising them to visit all stations to complete the passport), giving users immediate feedback and reinforcing engagement (by providing a stamp at the end of every station), and connecting children to support each other in working towards a common goal, gamification could have supported children's enjoyment and engagement with the initiative [30]. Additionally, the presence of the Captains likely reinforced this effect and supported the positive atmosphere, as previous studies have shown that the Captains can assist in reducing pain, boredom, and anxiety related to medical experiences for children and their families [31, 32]. Equally, the fun/gamification aspect of the event was perceived as balanced, appealing to the children and community while providing valuable health checks.

As previously mentioned, the community carries historical trauma from past externally led initiatives, meaning a positive and celebratory experience could have provided a corrective experience. Indigenous leaders have long advocated for more celebrations of Indigenous culture in Australia, such as with the annual National Aborigines and Islanders Day Observance Committee (NAIDOC) week, which honours the culture, history, and achievements of Aboriginal and Torres Strait Islander peoples [33]. To promote reconciliation, initiatives like NAIDOC week and the third birthday party are necessary.

Additionally, much of the health sector aimed at supporting Indigenous people is illness‐focused rather than prevention‐focused [23]. As a result, holistic health programmes that promote health and early intervention are recommended as best practice to support Indigenous people's health and well‐being [2, 23]. Thus, the birthday party event, targeted at early intervention and health education, was a proactive step forward towards a more prevention‐focused model of healthcare for Indigenous Australians.

Interestingly, a couple of participants noted that performing their health checks with children outside of the traditional clinic setting was beneficial for conducting better health checks. While outdoor or mobile health clinic screening studies have suggested benefits in overcoming logistical barriers and fostering more connection with local Australian communities, no systematic reviews have been published to date on whether conducting health screening for children in an outside environment is conducive to better health assessments [34, 35]. Thus, more research is warranted to confirm this effect.

### 4.5 Health Screening and Promotion

The event was perceived as an effective and holistic way to screen for health concerns for children living in Cherbourg. While the community has existing local health services, primarily provided through the Cherbourg Health Service, the event provided access to diverse specialist clinicians from various health disciplines who typically require travel to access. The health services were chosen in consultation with both the Cherbourg Health Service and Southern Queensland Rural Health, due to their specialisations in diagnosing and treating health conditions prevalent in Indigenous children and children living remotely, which facilitated efficient health assessments tailored to the unique needs of the community.

The logistical advantages of this approach are significant. Notably, Cherbourg is approximately a three‐hour drive away from central Brisbane, the main city in Queensland, meaning a considerable commute to central health services and infrastructure and signifying a significant burden to the community for accessing these services. Additionally, in remote areas like Cherbourg, specialist appointments for in‐clinic visits typically entail long wait lists and have the logistical burden of booking and attending appointments, which is exacerbated in rural Australia due to the limited local health team members and infrastructure [9, 20, 36]. The high throughput and breadth of diverse health stations involved in this initiative provided screening that would otherwise require multiple separate appointments across different locations and times. Consequently, we posit that an event like the third birthday party may have identified children in need of follow‐up earlier than if the event had not been held; however, this could not be confirmed with the data collected in this study.

The event served as a valuable opportunity to raise awareness among caregivers about age‐appropriate health expectations for children. Parents can accurately assess their children's developmental status, using tools like the Hearing and Talking Scale (HATS) and the Ages and Stages Questionnaire (ASQ‐TRAK), which have been validated in the Aboriginal and Torres Strait Islander cohort [37, 38]. However, research shows that caregivers living remotely may face additional logistical barriers to screening [37]. Therefore, helping inform caregivers of children's expected developmental milestones through the birthday party event removes the logistical barrier, empowering them to better assess their children's health and seek health services when needed. This strategy supports children's health, especially in rural areas with limited access to central health infrastructure and services.

However, the event's success in identifying health needs did not address a critical and widespread challenge in Australian paediatric rural health: the need for accessible and timely follow‐up care. The same geographical and logistical barriers that were overcome through the event still complicate ongoing treatment access. This transition from identification to treatment represents a significant gap that could potentially undermine the trust generated through positive engagement with Indigenous communities like Cherbourg. Without adequate follow‐up pathways, families may experience frustration and disillusionment, highlighting the need for sustainable care coordination and access beyond the initiative itself. Solutions for better coordination and access to follow‐up paediatric care in rural Australia require systemic healthcare solutions and highlight the need for government, not‐for‐profit, and community collaboration to ensure health programmes can achieve their full potential.

### 4.6 Engagement

Effective change requires strong community relationships built on ongoing commitment [23]. Research emphasises the imperativeness of ongoing positive interactions for trust and rapport‐building between healthcare providers and Indigenous communities, especially in light of traumatic histories with institutionalised services [9, 24, 39]. Many healthcare services provided to remote Australians are delivered by fly‐in‐fly‐out (FIFO) services, which were described as lacking sustainability by local Aboriginal communities due to the limited relationship‐building opportunities [9]. Accordingly, establishing an event like the third birthday party consistently could enhance attendance and trust. However, it is paramount that each iteration is systematically evaluated to adapt to the communities' evolving needs. Longitudinal studies could also offer insights into the long‐term impact on the community.

The desire expressed by participants to expand the event to other rural areas of Australia reflects a recognition of shared health concerns for children across the region. While this interest is noteworthy, it is essential to approach any replication of the event with careful ethical consideration. Specifically, plans to expand the initiative to other locations should be guided by principles of Indigenous self‐determination, respect for place‐based cultural knowledge and practices, and local governance structures; the success of the Cherbourg event was rooted in its co‐design approach and responsiveness to the specific cultural context, which cannot be simply copied and replicated to a different Indigenous Australian community. Recommending direct replication of the Cherbourg third birthday party in another Indigenous community risks overlooking the significance of place‐based knowledge, Indigenous authority, and community‐led decision‐making. Rather than viewing the Cherbourg model as a template, it should be viewed as an example of what is possible when programmes are grounded in strong local partnerships and culturally safe practices. Therefore, future efforts to recreate similar events in different communities necessitate a co‐design approach with those communities to meet their local needs and preferences [9].

Participants underscored the importance of stronger caregiver engagement to gain deeper insights into children's health and provide long‐term support. Extensive literature highlights the efficacy of family‐centred care in paediatric healthcare for Indigenous populations [40, 41]. In fact, caregiver health literacy and engagement in health services are strong predictors of children's health [42, 43]. As such, involving both the child and caregiver in health assessments may be more effective than engaging the child alone. Research shows that caregiver involvement in healthcare discussions not only improves children's health but also strengthens the partnership between caregivers and health professionals, promoting better health participation [44].

The suggestion to offer take‐away booklets and health strategies directly addresses the need for tangible resources that caregivers can utilise beyond the event. Research indicates that providing practical materials can reinforce health‐related information and support healthcare decision‐making, but theymust be relevant, readable, and understandable for its audience [45, 46]. For future events, health stations could produce a short take‐away booklet tailored to the community.

The suggestion to offer health checks for caregivers alongside children may promote greater participation and offer health benefits, but it must be carefully considered. Given the well‐established associations between social determinants of health and developmental outcomes for children, as well as the well‐known successful family systems approach to improving healthcare for minority groups, addressing caregiver health needs could potentially create stronger and broader improvements in family and child health [47, 48, 49]. However, logistical challenges, such as the increased health team members and resources needed to meet the added demand, need to be considered. Furthermore, health assessments for children and adults can vary drastically, posing additional challenges in terms of appropriateness and scope of the event; children's health assessments tend to focus more on developmental screening and monitoring based on their age, while adult screening may encompass assessments for chronic conditions or lifestyle‐related risk factors. As such, we posit that the third birthday party event may not be suitable for hosting such screenings. Alternative approaches, such as connecting families to existing family and/or adult health services, may be more appropriate.

The highlighted concern of an inconsistent flow of children visiting health stations during the event sometimes led to overcrowding at stations and resulted in inadequate time for health professionals to conduct thorough assessments. This could have resulted in some children receiving less attention than required, potentially affecting the thoroughness and accuracy of health assessments. Suggestions for future iterations could include streamlining the assessment protocols within a short time frame and implementing a set rotation system between health stations.

Lastly, more publicity for the event could have boosted the number of participants. While local radio and posters were used, more comprehensive multimedia strategies could have been employed to increase community engagement. Mailed invites, community newsletters, and partnering with local Elders, schools, and community organisations on other communication methods are considerations for future event iterations, especially “harder to reach” community members.

### 4.7 Health Checks

Ensuring an effective follow‐up referral process is important for tracking the children requiring further attention after a health promotion event. Hence, a streamlined and standardised protocol for referrals is vital. Additionally, participants expressed interest in conducting more in‐depth health assessments, which present challenges due to the fast‐paced nature of the event. As mentioned previously, health stations could pre‐plan a screening protocol that can be undertaken within the specified time constraints allotted for each child at the event.

### 4.8 Staff Workload

Healthcare coordination and access to follow‐up care represent a fundamental limitation that threatens the long‐term positive impact of initiatives like the third birthday party. The heavy workload of healthcare staff working in rural Australia is a common phenomenon [2], which poses challenges for recruiting health professionals to attend the event and for responding to follow‐up referral requests in a timely manner. This affects the breadth of services offered by future events and the quality of care the children receive. The inability to address referrals in a timely manner may also undermine the confidence the community has in the utility of the event and in the health system at large [2]. This jeopardises future community participation in similar initiatives and the trust essential for effective healthcare delivery in rural settings. Without other systemic and widespread improvements in the Australian paediatric rural system to ensure timely, quality healthcare for children living in rural Australia, this and other similar initiatives risk lacking positive long‐term effects for the community. This limitation must be recognised as a critical barrier that requires dedicated resources, systematic solutions, and likely improved models of care delivery to ensure that identification of need translates into accessible, timely intervention for children living in remote and rural Australia.

The lack of skilled healthcare professionals in rural Australia originates from ongoing difficulties in recruitment and retention, which intensifies pressure on the rural Australian workforce and increases the scope of practice for existing practitioners [2, 50]. Rural practitioners inherently manage a broader scope of practice across smaller populations, which is characteristic of rural healthcare delivery in Australia [2, 50, 51]. To alleviate some of this pressure, students could take on more of an active role in initiatives like the third birthday party, which could also support students' career development as culturally aware health practitioners [34].

## 5 Limitations

This study is not without limitations. Firstly, the findings are based on a single event, which may limit the generalisability of the results to other contexts, especially as other remote communities in Australia may have preferences and health needs that differ from Cherbourg. Additionally, the interview data relied on participants' recollection of the event, which may be subject to recall bias. Furthermore, the study did not measure the long‐term outcomes of the community's health status or health literacy, limiting the ability to evaluate the long‐term impact of the event.

## 6 Conclusion

The quantitative and qualitative data collected from the attendees indicated strong satisfaction and several benefits. It also highlighted potential future considerations for the event. Participants emphasised the need to identify and manage the health conditions of remote‐living Indigenous children and the importance of culturally sensitive healthcare provision, considering the traumatic historical experiences of Indigenous Australians. The event appeared to meet this goal, creating a safe and enjoyable medical experience for the community.

Community involvement via the opt‐in community‐led approach emerged as a prominent strength, exemplified by the positive community reception, high turnout, and caregiver involvement. Benefits were recognised also for healthcare staff and students alike, enhancing cultural competency and interconnectivity within the community and wider health system. The unique methodology was also well received; the holistic, prevention‐focused approach incorporating concepts of gamification, celebration, and the support of Starlight Captains contributed to the day's success and positive atmosphere and potentially encouraged community members to re‐engage in health services. The health assessments themselves were deemed useful in supporting children's health, especially given the challenges with remote communities in accessing health services. The event also provided a valuable opportunity to raise awareness of age‐appropriate health expectations of children, empowering caregivers.

Key considerations included administrative improvements, expansion and repetition of the event, stronger caregiver engagement, wider‐reaching publicity, and streamlined health station rotations and follow‐up processes. However, challenges in the heavy workload of remote‐working healthcare staff necessitate innovative approaches.

This study exemplifies a pivotal shift in addressing health disparities in remote Australian Indigenous children by incorporating culturally sensitive and fun elements into a healthcare initiative, promoting cultural competency in healthcare provision and education that can be applied to other programs in different Indigenous communities. Such initiatives not only enhance healthcare access but also serve as a model for improving health outcomes in traditionally underserved regions. Future research could seek to measure the long‐term impact and the effectiveness of implementing the described potential future considerations. A detailed capture and analysis of data regarding individual follow‐up appointments and interventions from each event to illustrate the direct impact of the program would be beneficial, as would a focus on understanding any factors that increase attendance and adherence, plus any further barriers to be addressed.

Overall, the Cherbourg third birthday party initiative highlights the importance of culturally appropriate early interventions in remote Australian Indigenous communities and provides valuable insights for future endeavours aimed at improving healthcare access and engagement for Indigenous children in Australia.

## Author Contributions

**Claire Treadgold:** conceptualisation, project management, data collection, analysis, writing – review and editing, supervision. **Erika Fortunati:** data curation, analysis, visualisation, writing – original draft, review and editing. **Rob Doyle:** data collection and curation, analysis, review and editing. **Jo Dann:** data collection, review and editing. **Aunty Kerrie Doyle:** methodology, analysis, writing – review and editing, supervision.

## Ethics Statement

We received ethics approval from Western Sydney University (HREC/2020/WSU/H11372).

## Conflicts of Interest

Claire Treadgold, Erika Fortunati, and Jo Dann are employed by Starlight Children's Foundation, which funded the study.

## Supporting information


**Data S1:** ajr70082‐sup‐0001‐DataS1.docx.

## Data Availability

The data that supports the findings of this study are available in the Supporting Information of this article.
