# Supplementary material for: Cake, Checkups, and Captain Starlight: Evaluating the Cherbourg Third Birthday Party Health Initiative for Children in Rural Australia
Source: Aust J Rural Health. 2025 Aug 7;33(4):e70082. doi: 10.1111/ajr.70082 (PMC12330202; doi:10.1111/ajr.70082)
Supplement: Supplementary file 1 — Data S1: ajr70082‐sup‐0001‐DataS1.docx. [file AJR-33-0-s001.docx]

**Table S1: Quotes illustrating the need and cultural considerations of the event**

| Theme | Subtheme | Illustrative quotes |
| --- | --- | --- |
| Need for the event | Health concerns | *“There is a massive gap in that age group where we kind of… I don’t like using the word “fail” … but we don’t tend to engage with them to encourage them to come in for regular health checks”*  *“When they come in at four years, we’re thinking, ‘Oh my goodness, if we only saw you last year or if we only saw you at this time, we might’ve got in sooner’”*  *“A lot of our children we felt were falling through the cracks”*  *“Some of these children are falling behind until it's almost too late”*  *“A lot of parents aren’t aware of what their children’s language should be at four years”*  *“Hearing health is a huge issue in this community”* |
|  | Support for parents | *“I think that’s the biggest thing is parents don’t have the support that I think they all need in today’s society”*  *“Cherbourg is such a young population that there’s more than a quarter of the population under 14 years of age, so there is a huge burden on the parents”* |
|  | Hope for the event | *“If we can at least get a bit of a feel for these kids at an earlier age, and maybe they might get checked, and there might be absolutely nothing wrong, but at least if we’re catching them a little bit earlier, we’re not always just trying to catch up.”* |
| Cultural Considerations | Celebration | *“We don’t celebrate enough”*  *“We want to see more celebrations in the community. We have had so much sadness and so much tragedy. It’s time that we celebrate these babies coming into this world, and it's time we celebrate them being in families”*  *“It’s been mentioned a few times by a couple of our elders in the community - that something they would love to see is a lot more celebration. So, I’m hoping that’s the beginning of a lot more to come”*  *“It was cool that we had a cake because it was a lovely celebration of what’s there now, what they’re doing. And it was something to be able to give to the people that came all the way out and were there for a long time”* |
|  | Importance of community connection and representation | *“I think it’s imperative to have that really strong connection with the clinic or a hospital like we had”*  *“The other thing that worked was that it was as local as we possibly could make it. There was very little that we brought in from Brisbane. It was a very local event”* |

**Table S2: Quotes illustrating the strengths of the event**

| Theme | Subtheme | *Illustrative Quotes* |
| --- | --- | --- |
| Community involvement | Autonomy | *“We increase that awareness, but we make the community feel comfortable and make sure the community knows that it’s their decision [to be involved] … I think it’s taking the pressure off anyone within the community to be like “we HAVE to come along” so, it made it more fun”*  *“It’s in their environment, and it’s their choice”*  *“We had community members playing music, and we had speeches from community members, and I think it was just like it showed a nice sense of unity like we both working towards the same thing rather than just a push onto the community of you should be doing this, and you should be getting your child checked for this kind of thing”* |
|  | Positive reception from the community | *“It was a positive reception from the community”*  *“They (the community) were really excited about it actually. They could really see that this was something that would be enticing for families”*  *“A couple of elders in the community were there, and it felt very community centred”*  *“It was a great community adventure, and I think there were a lot of people that got a lot out of it”* |
|  | Caregiver involvement | *“There were a lot of like adults who got involved this time. I think usually, when I go on other trips, the kids are really into it, and the adults are a bit more apprehensive. But it felt like, at this event, like the adults were more relaxed because we were outside, and it was more of a fun environment than being in the waiting room”* |
|  | High turnout | *“I thought it was a fantastic turnout for the community”*  *“We felt like there was good turnout compared to an outpatient service, where you find a lot of people not turning up”*  *“If there’s a great turnout, then we, as the people servicing them, are very highly motivated to continue engaging with this community. I suppose that’s really beneficial for the long term if we were to set up any projects or expand our services more in that area”* |
|  | Benefits to staff | *“It’s been very rewarding”*  *“It was really nice to engage with clinicians that we haven’t necessarily seen before”*  *“It was about community engagement, getting to know the families and networking between service providers themselves. Building on that network and knowing that our clinicians are able to seek advice or refer to other existing services”*  *“Having a chance to meet the elders that came was awesome as well”*  *“Getting to know the community is probably the first thing. Just getting to know them and getting to mingle with the families and people around there is probably the biggest effect for a clinician”* |
|  | Benefits to students | *“It was a great way for students to see families in a different environment and apply their skills in a non-clinical environment”*  *“From my perspective, it's absolutely essential that we’re actually getting students to become a bit more aware of the communities and how to work within those remote areas. Particularly Aboriginal and Torres Strait Islander children”*  *“For the students that went, I think it was great to be engaged in some sort of health promotional activity like that, but they rarely get the opportunity to see kind of, you know, to see many Indigenous clients”* |
|  | Teamwork | *“It was just amazing how everyone rallied together, and I think that was probably why it worked so well”*  *“I was just blown away by how everyone was just so keen to help out, everyone was just so positive about the day, and it just had an amazing atmosphere”* |
| Methodology | Birthday party concept | *“Taking it outside of my clinic room and into the yard was probably the nicest thing about it… its very different to the hospital environment, and when they come here, the kids feel that it’s not quite that scary”*  *“It was a good way to theme it and make it a fun day but also getting the outcome of the kids having their health check”*  *“It was nice and balanced - a nice mix of games and serious tests”* |
|  | Facilitating a positive atmosphere | *“Because there was food, music, prizes, Captains, and a whole bunch of kids, it just turned into something really fun, and some of the kids started making friends and seeing if they could get to each station, which was really sweet”*  *“I think they (the children) enjoyed the vibrant atmosphere Starlight created, like parties and cake and dancing. I think children genuinely enjoyed it”*  *“So, it was just lovely to see the smiles of young children that you know going through a wellness check”* |
|  | Health passport concept | *“They had the little stamps in the passports. And we thought that was great because the parents were motivated because they knew they wanted their kids to have a goodie bag at the end”*  *“It was a nice little incentive for them to go around and visit each station and interact with every aspect of the event”* |
|  | Better health assessments | *“In that sort of more fun setting, it probably captured a better picture of where their skills are at compared to when we bring them in a one-on-one clinic setting that might be a bit foreign to them”*  *“There’s always going to be struggles in a group setting where you might get distractions but then you can also see their social skills and how they interact with their peers and those negotiation skills and their sharing or taking”*  *“Physio-wise, I think it was a great way of screening kids”* |
|  | High throughput | *“It’s so great to see so many kids getting early intervention health checks and in a really quick way. It was like rapid fire. Because when they go to the clinics, they could be there forever. You know? There can be such hectic wait times just to see like one thing, where this was like ‘we're going to cover all these things in 20 minutes’. I think that was incredible”*  *“A lot of the health professionals were saying there they were glad that they saw so many kids”* |
|  | Captain Starlight | *“Captains are just amazing. They don’t take long, and those kids are so relaxed”*  *“The kids are so receptive. They just love it. And they love to join in. It’s good to see. Usually, they hide behind everyone else, but the Captains come out, and they go, ‘oh look at this, a crazy person wearing a suit!’”*  *“The kids just wanted to talk and interact with them... It meant that we could actually just sit back and watch them interacting, which gave us as much information, probably more information, than if we were in there trying to interact with the kids”* |
| Health screening and promotion | Children's health | *“A lot of kids got through and got a lot of health checks. I think it’s a huge benefit and a huge health impact”*  *“I think there probably will be some earlier referrals than when the child has their 4-year check-up”*  *“She (the organiser) did keep all the passports, so she was going to work through them and see those that the clinicians that might’ve written in there to say you need to have a check-up in a month. So that’ll really help her to create a database with that to know when they should be contacting these families and who they needed to see again”* |
|  | Caregiver health education and awareness | *“It has made more parents aware of what other things we do”*  *“These types of things mean that the families will have more awareness of what things to be looking for and when to be concerned. So they can not only just monitor that child but also their siblings. I would like to think that, hopefully, this type of thing means that everyone within the community is more aware of the health services”* |
|  |  | *“I wasn’t really able to get a whole lot of information on the day from the parents, but I think it was still nice. To still have that sort of face-to-face contact to show this is who we are, this is what we do, and this is how we can help”*  *“I actually did not come across anyone who we had to follow up from a physio point from that session, but I think it was a good one to involve the community and make them aware of what health services are about”*  *“They get to know what hospital is about. It's not just always about medicine and injections and those sorts of scary things. I’m hoping that once they come to a function like this, we will start to see more attendance rate to appointments”* |
|  | Improving the relationship between the community and healthcare professionals | *“It also gave families and kids an opportunity to see how much fun they could have with medical professionals... they thought it was great, and some of the kids didn’t want to go home”*  *“Getting the kids going to these medical facilities which people in the community can be sceptical of for a number of reasons and being more comfortable and knowing that it’s not like a big scary deal… and then they are more likely to want to come back and visit when they do turn four or when their next check-up is, and not stigmatising for them”*  *“It provided just like a safe space for some healthcare. From my experience and from what I’ve heard, they don’t necessarily like going to the clinics, but when we’re (Captains) there, I feel like it turns into more of a fun sort of opportunity”* |
|  | Returning for health appointments | *“Some of the parents have come saying they are more happier to bring their kids back”*  *“I reckon there would be people looking out for it next year”*  *“I noticed there was feedback where (the parents) were saying “I didn’t know if I was going to come today,” they were a bit sceptical of the whole third birthday party thing, and then afterwards they said, “This is great, were really glad that we came”* |
|  | Understanding the health needs of the community | *“There weren’t any formal referrals to our service, but it gave us an idea of what the needs were in the area. It gets us to think about whether we can provide more of a service if it's not one-on-one or what other things we can do to help”*  *“Understanding what the community needs and understanding the caseload and how we can help and figuring out how that’s going to fit into our schedules”* |

**Table S3: Quotes illustrating the future considerations and improvements**

| Theme | Subtheme | Illustrative Quotes |
| --- | --- | --- |
| Administrative | General comments | *“I thought it was such a well-planned event”* |
|  | Check-in process | *“I think the registration process was a little bit slow”*  *“A better check in check out process”* |
|  | Layout | *“A map of who’s going where on the day, who’s marquee is going to be where… would just be helpful”*  *“When we got there, we didn’t really have a plan for how we were going to set up. So, it was kind of a bit of a rushed”*  *“A few of the stalls’ set-up kind of blended a little bit. So, for example the dietary stuff and the dentist were very close together, or they were under the same banner, and that was kind of confusing for us Captains”*  *“We were beside the occupational therapists, so we had that bouncing between the two tables, which worked really well because, obviously, we sort of look at the same things”* |
|  | Location | *“There could be a different space around the hospital to do it because it was tucked around the side. They had to know it was on to attend the event; it wasn’t quite a great foot traffic area… if people can see you, then they’re more inclined to come over and be like, “What’s going on here?”*  *“Does it need to be near the hospital? Could it be like somewhere else in the community that could have, like, higher foot traffic or something?”* |
|  | Signage | *“We had a little sign that was dietetics, but I think some bigger, better signage … I don’t know that we all had great signage”*  *“I think that signage and visual messaging can be more effective”*  *“Ours was an apple or something, but I think someone said ours was a weird sign you didn’t know what it was”* |
|  | Timing and heat | *“It was just unbearably hot, and I think that might have kept a lot of families away”*  *“Everyone was having a good time like the kids didn’t want to leave, which is always a good sign. But it was kind of the heat of the day kind of slowed things down, and I think just like crushed a little bit of potential because a few kids, you know, they kind of go to 2 or 3 stations, have a good time, and then their parents or their leaders are ushering them out”*  *“It was an extremely hot day. So a different time of year or time of day would be probably something to think about”*  *“I think if we could do it maybe like earlier in the day or later in the year. It would be better, and people would be willing to stay longer because they’re not sweltering”*  *“They ended up sheltering under other tents that were part of the health professionals, and they got really crowded, then they started moving on. So maybe a few more tents, maybe just shade sections to share”* |
| Engagement | Building rapport | *“It does take you a while to build up that rapport with kiddies, and they look at you and think ‘ohh’, but once you sit down and start playing, they’re good”*  *“We don’t have established relationships, so a few of them didn’t really know we were a trusted person”*  *“Building that relationship with the community to let them know that hey, we’re here, we’ve got these services available for your children”* |
|  | Repeating the event | *“If it’s an annual event, it becomes ingrained in the community that it’s going to happen”*  *“I would encourage you to keep going”*  *“If it is done yearly, I think it's something that the community can look forward to, and you’ll get more and more stalls out there, and the event will become bigger and bigger and even better for all the kids and for all the families in the community”* |
|  | Expanding the event | *“I certainly think that there’s a lot of other communities that are quite interested in this sort of style”*  *“I think this (health concern) is quite common across all health services. It’s not just common to Cherbourg”*  *“There are a number of other Aboriginal and Torres Strait Islander communities across Queensland, if not nationally, that could benefit from this”*  *“We’ve even had interest from a community out past Cherbourg – their medical service is interested in a similar model”* |
|  | Caregiver engagement | *“I just feel like we should target the adults because the adults are the ones deciding what goes on the plate… the kids will eat what the parents are eating”*  *“They probably need those strategies hand in hand with how to support the kids”*  *“There was a suggestion that with the parents and grandparents' participation, maybe they too can have a health check because they too often neglect their own health and wellbeing…also give them a little gift as an appreciation for participating and bringing their child. I know the main focus is on the 3-year-olds, but sometimes it's nice to reward the parents for participating and giving them the opportunity to have a health check as well”*  *“It would be useful to have more of that family engagement… I guess someone who can actually tell us more about what that child is like at home and those sorts of things”* |
|  | Time with children | “*Sometimes we had a lot of children, and sometimes we didn’t really have any children around”*  *“They all kind of flocked to the table so I didn’t feel like it was a really great opportunity to actually talk to the kids and try and get that informal assessment. I wasn’t really able to get a good picture of how they presented”*  *“I wish I were able to spend more time with the one child particularly, but I wasn’t able to”* |
|  | Publicity | *“I’d like to see more publicity for it next year”*  *“It’s just good to even plan for next year and invest some time and event management and promotions not just for the service providers but also for the community; what is the best multimedia approach that we can take to encourage parents and those hard-to-reach community members?”* |
| Health Checks | Follow-up referral process | *“We knew that there would be a lot of referrals from it, and that’s probably been the challenge, is to be able to recall them”*  *“We just want to make sure that there’s follow-on and follow-up, rather than just an event that exists once and then there’s no actions or activities that come from it”*  *“I just feel we probably need to be a bit more strategic in what we're doing… what’s the follow-up going to be rather than just going out to be there… just that streamlining”* |
|  | More in-depth health checks | *“Hearing was difficult to do what wanted to do. I think definitely when you look in their ears, it’s an achievement, but then don’t even bother looking at audiology, which is a shame”*  *“I feel from the dietetics side we could have more influence…. because even for our profession, they were getting checked by the nurses for their height and weight. And so that was the check, but we weren’t doing any kind of food assessment, but potentially, we could have”*  *“From a dietetic point of view, we weren’t willing to do that (a formal assessment) because that meant a clinical event and so if we did that, we would have to write about 80 referrals and then class that as an occasion of service. We don’t have the capacity to do that”*  *“I think with speech pathology, to expect a three-year-old to talk to a complete stranger within like 5 minutes of meeting them kind of thing. So that was probably our challenge”*  *“If there could be some identified people to do a brief overview assessment. A questionnaire maybe”* |
|  | Healthier foods | *“We thought maybe if there was carrot cake? because one of the messages that we would give would be to just put vegetables in everything you can think of. Snack on it. Eat it for breakfast. Eat it for lunch. Eat it for dinner. Cook it, raw. It doesn’t matter”* |
| Staff workload | Timeliness of follow-ups | *“It will have an impact on our service with workload”*  *“it’s impacted the service to be able to recall those children and actually get them through in a timely manner, so probably looking at getting children that will be going to kindy next year first and then working back. It’s probably just time management and having the staff do that”* |
|  | Lack of resources | *“Our dietician said there’s no room for more follow-up. Even if we did highlight there’s no capacity to see more people”*  *“We didn’t stay for most of the party because of other clinical demands. To be honest, we were understaffed at the time, so the event wasn’t our priority”*  *“It depends on the clinical demands on our staff as well at the time. So often in rural areas, we’re not always fully staffed. I think it just depends on our schedules and our demands and whether we are able to allocate clinicians of all disciplines to be all out on the same day”*  *“Having to take that time out of your clinical workload was a challenging thing.”* |
|  | More staff | *“I think it’s something students could do; I do think it’s something that doesn’t necessarily require a clinician”*  *“We were going to get somebody else to set up *laughs*. Providing the service during the day, we just, we were flogged”*  *“Having it become a student-led activity so we’re helping upskill new healthcare professionals in working with the Indigenous population, but also it takes that burden off the clinicians that have a heavy workload”*  *“I would even suggest maybe a couple more captains. I mean, it’s hard to predict how many kids are going to show up, but there were so many kids, and I think we could have gotten more kids to each station if we had more Captains”* |

Interview Questions

1. Can you tell me about your experience with the Cherbourg 3^rd^ birthday party and how you were involved?
2. What do you perceive as the benefits/impact of the event? (*prompt: for children, caregivers, staff, healthcare system, long-term/short-term*)
3. What helped make this event successful?
4. What could be improved should this type of event be run again?
5. Would you recommend that this event be run again in the future?
